# Bone marrow-derived mesenchymal stem cells in three-dimensional co-culture attenuate degeneration of nucleus pulposus cells

**DOI:** 10.18632/aging.102390

**Published:** 2019-10-30

**Authors:** Xunlin Li, Aimin Wu, Chen Han, Chen Chen, Tangjun Zhou, Kai Zhang, Xiao Yang, Zhiqian Chen, An Qin, Haijun Tian, Jie Zhao

**Affiliations:** 1 Department of Orthopaedics, Ninth People’s Hospital, Shanghai Jiaotong University School of Medicine, Shanghai Key Laboratory of Orthopaedic Implants, Shanghai, P. R. China; 2 Department of Spine Surgery, Zhejiang Spine Surgery Centre, Orthopaedic Hospital, The Second Affiliated Hospital and Yuying Children’s Hospital of the Wenzhou Medical University, The Second School of Medicine Wenzhou Medical University, The Key Orthopaedic Laboratory of Zhejiang Province, Wenzhou, P. R. China

**Keywords:** nucleus pulposus, senescence, bone marrow-derived mesenchymal stem cells, 3D co-culture, ZMPSTE24

## Abstract

Intervertebral disc degeneration (IDD) is an irreversible aging-associated clinical condition of unclear etiology. Mesenchymal stem cells (MSCs) have the potential to delay IDD, but the mechanisms by which MSCs attenuate senescence-related degeneration of nucleus pulposus cells (NPCs) remain uncertain. The present study employed a three-dimensional (3D) co-culture system to explore the influence of MSCs on NPC degeneration induced by TNF-α in rat cells. We found that co-culture with bone marrow-derived MSCs (BMSCs) reduced senescence-associated β-galactosidase expression, increased cell proliferation, decreased matrix metalloproteinase 9, increased Coll-IIa production, and reduced TGFβ/NF-κB signaling in senescent NPCs. In addition, expression of zinc metallopeptidase STE24 (ZMPSTE24), whose dysfunction is related to premature cell senescence and aging, was decreased in senescent NPCs but restored upon BMSC co-culture. Accordingly, ZMPSTE24 overexpression in NPCs inhibited the pro-senescence effects of TGFβ/NF-κB activation upon TNF-α stimulation, while both CRISPR/Cas9-mediated silencing and pharmacological ZMPSTE24 inhibition prevented those effects. Ex-vivo experiments on NP explants provided supporting evidence for the protective effect of MSCs against NPC senescence and IDD. Although further molecular studies are necessary, our results suggest that MSCs may attenuate or prevent NP fibrosis and restore the viability and functional status of NPCs through upregulation of ZMPSTE24.

## INTRODUCTION

Intervertebral disc degeneration (IDD) is a widespread condition in the aging population, frequently triggered by premature senescence and dysfunction of intervertebral disc cells [1]. Because of the pivotal role of intervertebral discs in supporting the normal function of the vertebro-spinal axis [2], disc degeneration can accelerate normal spinal aging and cause severe neck and back pain [3]. Following terminal differentiation, cells in high-load structures such as intervertebral discs face higher senescent stress than cells in other motor organs; factors at play include cell aging, stem cell depletion, and protein homeostasis imbalances, among others [4]. Thus, compounded by increased longevity, the prevalence of IDD diseases such as cervical spondylosis, lumbar disc herniation, and discogenic low back pain has increased over the years to become the main cause of life’s quality decline in the elderly [5, 6].

As the core constituent of intervertebral discs, the integrity of the nucleus pulposus (NP) and its associated proteoglycan mucoid-like matrix is essential to withstand compressive loads [7]. To address the high burden of IDD and related conditions, many studies have focused on maintaining the number of nucleus pulposus cells (NPCs) and enhancing the deposition of functional components of the extracellular matrix (ECM) [8]. To this end, the potential of mesenchymal stem cells (MSCs), represented as progenitor NPCs in the NP, has been intensively explored in the field of IDD research [9, 10, 20]. Whereas in intact discs the annulus fibrosus that surrounds the NP limits the differentiating potential of MSCs [11], in vitro experiments showed that activated MSC clusters react to pro-inflammatory stimuli by releasing anti-inflammatory factors [12, 13] and influence paracrine coordination of cell cycle, to delay or even reverse aging at both cellular and organismal levels [14]. Indeed, research has shown that MSCs can influence NPC survival and function through paracrine effects involving the release of soluble cytokines and extracellular vesicles [15–17]. Investigations also showed differential NP gene expression in IDD models in response to stress-related processes such as aging [18], oxidation [19], the unfolded protein response [20], and metabolism [21].

The ZMPSTE24 gene encodes a zinc metalloproteinase involved in the endoproteolytic processing of prelamin A to mature lamin A [22]. Mutations in the ZMPSTE24 gene give rise to several laminopathies, reproduced to a large extent in ZMPSTE24-deficient mice. These include neonatal lethal restrictive dermopathy, premature aging disease (Hutchinson-Gilford progeria syndrome), and mandibuloacral dysplasia [23, 24]. During aging or oxidative stress, ZMPSTE24 protein activity is reduced, leading to abnormal cell mitosis and DNA damage repair and early-onset cellular senescence [25, 26]. Research further showed that ZMPSTE24 exerts quality control of protein synthesis in the endoplasmic reticulum, protecting cells against proteotoxicity by cleaving fragments of clogged proteins at translocon sites [27, 28].

Our research group has studied the therapeutic effects of MSCs on mammalian NPC degeneration [29]. The purpose of this study was to investigate whether bone marrow-derived MSCs (BMSCs) in three-dimensional (3D) co-culture can relieve senescence features in NPCs. To this end, and following assessment of human NPCs in clinical IDD samples, we used in vitro and ex-vivo inflammatory senescence NPC models to explore the involvement of pro-fibrotic (i.e. TGFβ) and pro-inflammatory (i.e. NF-κB) signaling pathways, and the potential role of ZMPSTE24 in restoring the functionality of senescent NPCs.

## RESULTS

### MSC frequency correlates with intervertebral disc degeneration in humans

**Figure 1:**
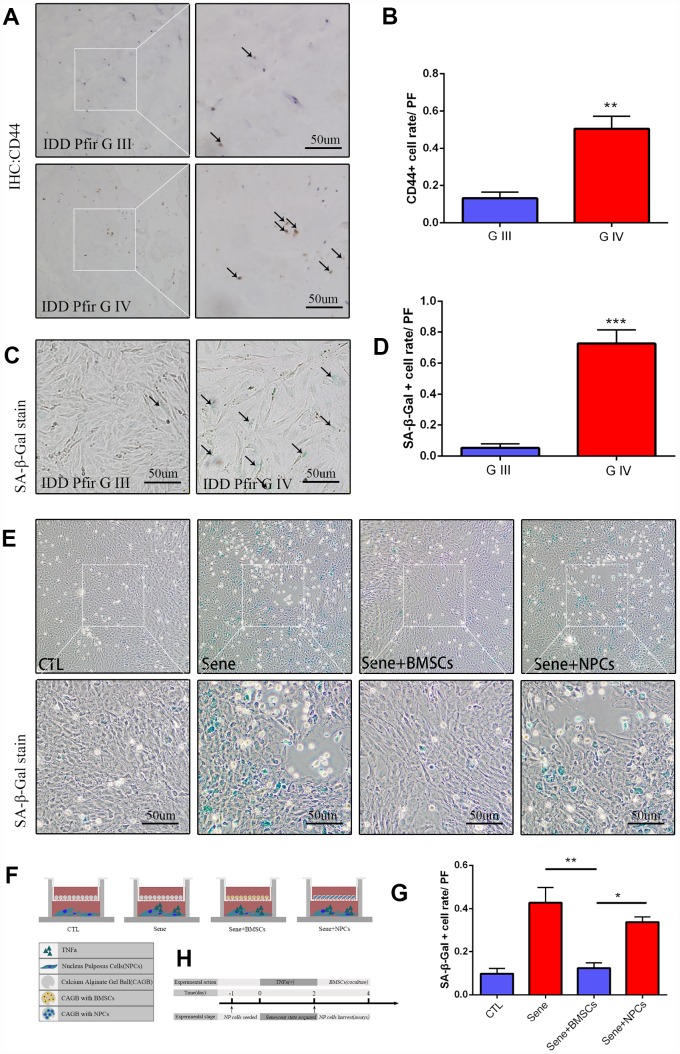
**Mesenchymal stem cells activated in IDD, and cocultured BMSCs can alleviated the nucleus pulposus cells senescent rate in vitro.** (**A**) Immunohistochemistry staining of CD44 in intervertebral disc degeneration of Pfirrman grade III(G III) and grade IV(G IV). n=5, Scale bar, 50um. (**B**) CD44 positive cell rate between G III and G IV were determined by using Image J software. (**C**) SA-β-Gal staining of primary NP cells of IDD Pfirrman grade IV and Pfirrman grade III. n=5, Scale bar, 50um. (**D**) SA-β-Gal positive cell rate between G III and G IV were determined by using Image J software. (**E**) 3D coculture models of were established as schematic diagram described. SA-β-Gal staining of senescent NP cells after 2 days coculture of normal NP cells +blank calcium alginate gel balls(CTL), senescent NP cells+ blank calcium alginate gel balls(Sene), senescent NP cells +calcium alginate gel balls with BMSCs(Sene +BMSCs), senescent NP cells +calcium alginate gel balls with normal NP cells(Sene +NPCs), n=3, Scale bar, 50um. (**F**) Co-cultivation pattern design and experimental groups. (**G**) SA-β-Gal positive cell rate between CTL, Sene, Sene +BMSCs and Sene +NPCs groups were determined by using Image J software. (**H**) Experimental flow diagram Values represent means±S.D. Significant differences between different groups are indicated as *P < 0.05, **P < 0.01, ***P < 0.001. PF: per field.

Activation of resident or recruited stem cells characterize the regenerative response to tissue senescence or damage. To evaluate the presence of MSCs in clinical NP specimens, Pfirrmann grade III (G III) and IV (G IV) discs were subjected to immunohistochemical staining to detect CD44 [30]. Results showed that MSC-specific CD44 expression was significantly higher in G IV than in G III samples (Figure 1A and 1B). Next, primary NPCs were extracted from G III and G IV discs, cultured, and senescence-associated β-galactosidase (SA-β-Gal) staining was performed on passage 1 cells. Results showed that the number of SA-β-Gal-positive NPCs was significantly higher in G IV than in G III cultures (Figure 1C and 1D).

### Co-culture with BMSCs attenuates NPC senescence

To explore the regulatory effects of MSCs on NPC senescence, rat BMSCs were complexed with 2% sodium alginate to generate 3D Calcium Alginate Gel Balls (CAGB) cultures (Supplementary Figure 1B). Senescence was induced in cultured rat NPCs by pre-treatment with 20 ng/ml TNF-α for 48 h, and these cells were then added to the lower chamber of the co-culture pool (Figure 1F, Supplementary Figure 1A). SA-β-Gal staining revealed that the rate of senescence was significantly lower in NPCs co-cultured with BMSCs (Sene + BMSCs) compared to both senescent NPCs co-cultured with cell-free CAGB (Sene) or CAGB complexed with normal, non-TNF-α-treated NPCs (Sene + NPCs; CTL) (Figure 1E and 1G).

### BMSCs promote Coll-IIa expression and decrease MMP9 synthesis in senescent NPCs

**Figure 2:**
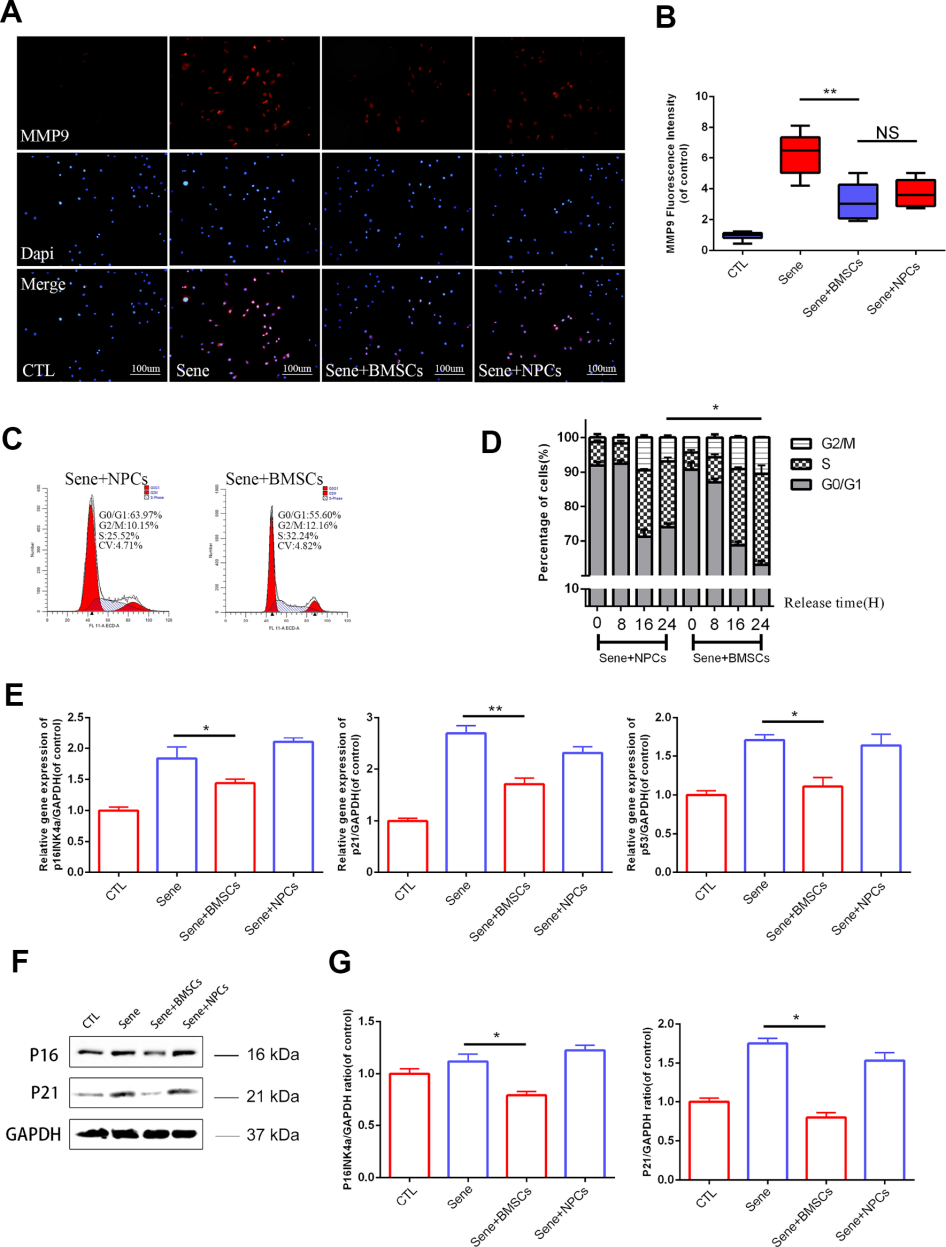
**Senescent NPCs’ ECM degradation and cell cycle suppression been alleviated in BMSCs coculture model.** (**A**) Immunofluorescence staining of MMP9 combined with DAPI staining for nuclei. n=5, Scale bar, 50um. (**B**) MMP9 fluorescence intensities were determined by using Image J software. (**C**–**D**) Flow cytometry results between sene +BMSCs and sene +NPCs. n=3 (**E**) The mRNA expressions of p16Ink4a, p21 and p53 were measured by real time PCR. n=3. (**F**–**G**) The protein expressions of P16, P21 were visualized by western blot and quantified by Image J. Values represent means±S.D. Significant differences between different groups are indicated as *P < 0.05, **P < 0.01.

Senescent NPCs show impaired synthesis of extracellular matrix (ECM) components, such as Col-IIa, and enhanced production of proteins involved in ECM breakdown, such as those in the metalloproteinase (MMP) family. Immunofluorescence evaluation of NPCs from various CAGB co-cultures showed that MMP9 expression was significantly lower in the Sene + BMSCs system compared to the Sene group, but did not differ from that seen in Sene + NPCs co-cultures (Figure 2A and 2B). Meanwhile, Col-IIa immunofluorescence was significantly higher in NPCs in the Sene + BMSCs condition than in NPCs in Sene cultures, and comparable to that seen in the CTL group (Supplementary Figure 1F and 1G). These data indicate that the presence of BMSCs restored the ability of senescent NPCs to balance the deposition and degradation of ECM components. Also, since MMP9, but not Col-IIa, expression in senescent NPCs in the Sene + NPCs group was comparable to that observed in Sene + BMSCs co-cultures, we surmise that normal NPCs can partially attenuate the catabolic action of senescent NPCs on ECM, but do not affect their impact on ECM anabolism.

### BMSCs restore the viability of senescent NPCs

We next assessed whether BMSCs could improve the viability and replicative potential of senescent NPCs. Assay results showed that both cell viability (measured through the CCK8 assay) and colony formation ability were improved by co-culture with BMSCs (Supplementary Figure 1C–1E). To evaluate these effects in more detail, flow cytometry was used to analyze cell cycle distribution in senescent NPCs from different co-cultures. Results showed a larger proportion of NPCs in active S phase in Sene + BMSCs (26.43% ± 2.40%), compared to Sene + NPCs (19.11% ± 1.23%) (Figure 2C and 2D).

### BMSCs downregulate cell cycle inhibitory genes in senescent NPCs

To further verify the effect of BMSCs on cell cycle check-point activation in senescent NPCs, q-PCR was used to detect the transcriptional expression of p16^Ink4a^, p21, and p53. The expression of all these genes was significantly lower in NPCs from Sene + BMSCs compared to Sene co-cultures (Figure 2E). Parallel western blot analyses confirmed that p16^Ink4a^ and p21 proteins were downregulated in NPCs from Sene + BMSCs co-cultures (Figure 2F and 2G), while p53 expression did not change (data not shown).

### BMSCs reduce the senescence-associated secretory phenotype of NPCs by upregulating ZMPSTE24

**Figure 3:**
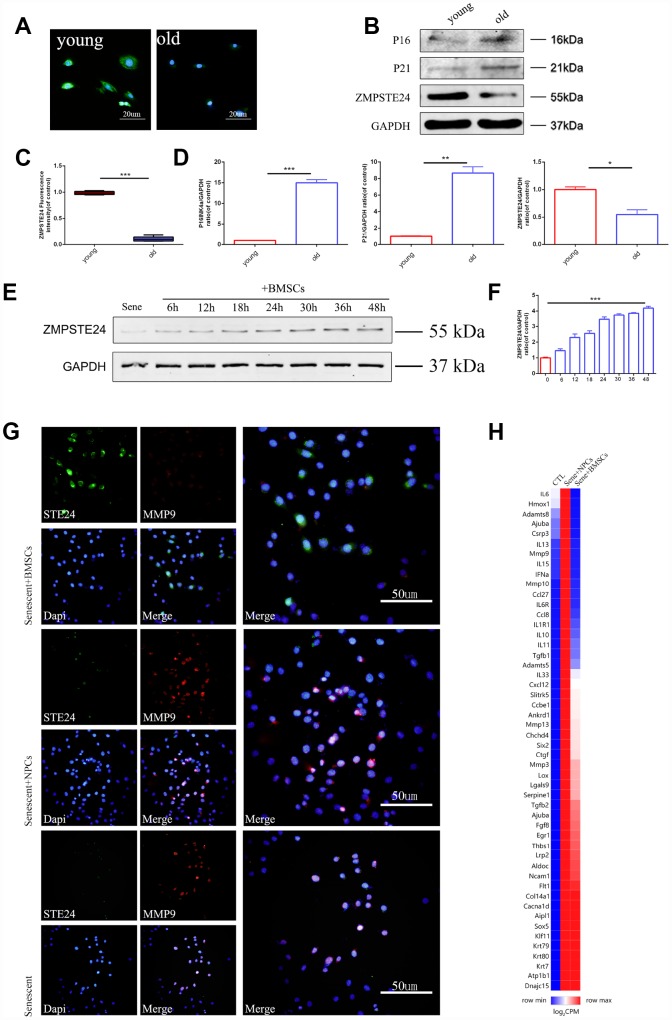
**3D BMSCs coculture activates ZMPSTE24 signaling.** (**A**–**C**) Immunofluorescence staining of ZMPSTE24 between young (3 weeks) and old (24 months) rats, scale bar, 50um. Fluorescence intensities were determined by using Image J software. (**B**–**D**) Protein expression of P16, P21 and ZMPSTE24 were visualized by western blot and quantified by Image J. (**E**–**F**) Protein expression of ZMPSTE24 during coculture was visualized by western blot and quantified by Image J. (**G**) Immunofluorescence staining of ZMPSTE24 and MMP9 in NP cells between Sene, Sene +NPCs and Sene +BMSCs groups. n=5. Scale bar=50um. (**H**) Heat map representation of SASP (senescence-associated secretory phenotype) genes between CTL, Sene +NPCs and Sene +BMSCs groups. n=3 biological replicates. Values represent means±S.D. Significant differences between different groups are indicated as *P < 0.05, **P < 0.01, ***P < 0.001.

Age- and mutation-dependent reductions in the activity of the zinc metallopeptidase ZMPSTE24 compromise cellular DNA repairing mechanisms and are linked to organ disfunction and premature cell senescence. ZMPSTE24 expression analysis in rat intervertebral discs showed age-related downregulation (Figure 3A–3D), while co-culture with BMSCs reactivated ZMPSTE24 regulatory activity in senescent NPCs (Supplementary Figure 3). Western blotting further showed that ZMPSTE24 expression increased over time in NPCs co-cultured with BMSCs (Figure 3E and 3F). Co-localization assessment of ZMPSTE24 and MMP9 by immunofluorescence indicated that ZMPSTE24 was upregulated in NPCs from Sene + BMSCs co-cultures, but not in NPCs co-cultured with normal NPCs (Sene + NPCs) (Figure 3G). Meanwhile, MMP9 expression was significantly downregulated in NPCs from Sene + BMSCs co-cultures, compared with both the Sene and Sene + NPCs groups (Figure 3G). We subsequently performed RNA sequencing (RNA-seq) to compare gene expression between NPCs grown in Sene + BMSCs and the Sene + NPCs co-cultures. Results demonstrated that co-culture with BMSCs induced a global reduction in the senescence-associated secretory phenotype (SASP), represented by several pro-inflammatory cytokines, proteases, and growth factors, in senescent NPCs (Figure 3H).

### ZMPSTE24 overexpression inhibits RelA nuclear translocation and attenuates NPC senescence

**Figure 4:**
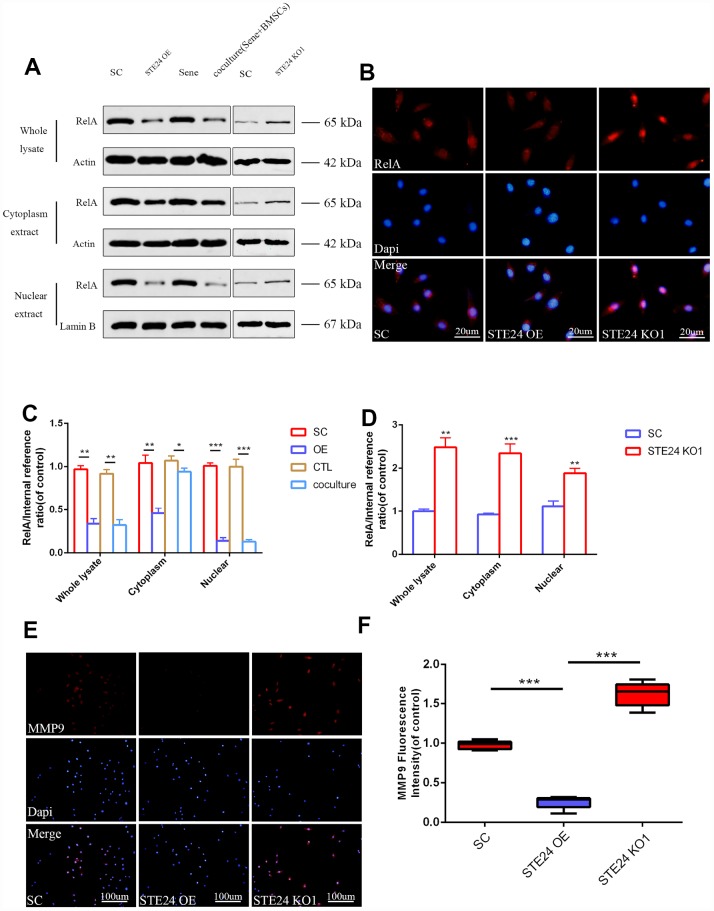
**Correlation between ZMPSTE24 and TNF-α induced RelA activation.** (**A**, **C**, **D**) Protein expression of RelA with stable empty vector (SC), ZMPSTE24 overexpression or knockout of ZMPSTE24 were visualized by western blot and quantified by Image J. n=3. (**B**) The nuclear translocation of RelA was detected by immunofluorescence combined with DAPI staining for nuclei. n=5. Scale bar: 20 μm. (**E**–**F**) Immunofluorescence staining of MMP9 combined with DAPI staining for nuclei. n=5, Scale bar, 50um. Values represent means±S.D. Significant differences between different groups are indicated as *P < 0.05, **P < 0.01, ***P < 0.001.

To explore the effects of ZMPSTE24 on inflammation-induced NPC senescence, we designed three sets of CRISPR/Cas9 vectors (KO1-3) to inactivate the rat ZMPSTE24 gene (Supplementary Figure 2A), as well as an ZMPSTE24 overexpression (OE) plasmid (Supplementary Figure 2B). After stable transfection and stimulation with TNF-α (20 ng/ml), NPCs’ whole cell extracts, as well as cytoplasmic and nuclear fractions, were analyzed by western blotting to assess the expression levels of RelA/p65, a protein required for NF-κB heterodimer formation, nuclear translocation, and activation (Figure 4A–4D). Results showed that ZMPSTE24 OE dramatically mitigated nuclear translocation of RelA induced by TNF-α (Figure 4B). In addition, immunofluorescence showed significant inhibition of MMP9 expression in TNF-α-treated NPCs overexpressing ZMPSTE24 (Figure 4E and 4F), compared to both scrambled vector (SC) control and ZMPSTE24 KO1 cells. Moreover, fluorometric SA-β-Gal (SA-Spiderβ-Gal) staining in living NPCs showed a significantly lower senescence rate in ZMPSTE24 OE compared with SC control cells. Meanwhile, the rate of SA-β-Gal-positive cells was higher in ZMPSTE24 KO1 than in SC NPCs, although the difference was not significant (Supplementary Figure 2C and 2D).

### BMSC-induced ZMPSTE24 upregulation decreases TGFβ signaling in senescent NPCs

**Figure 5:**
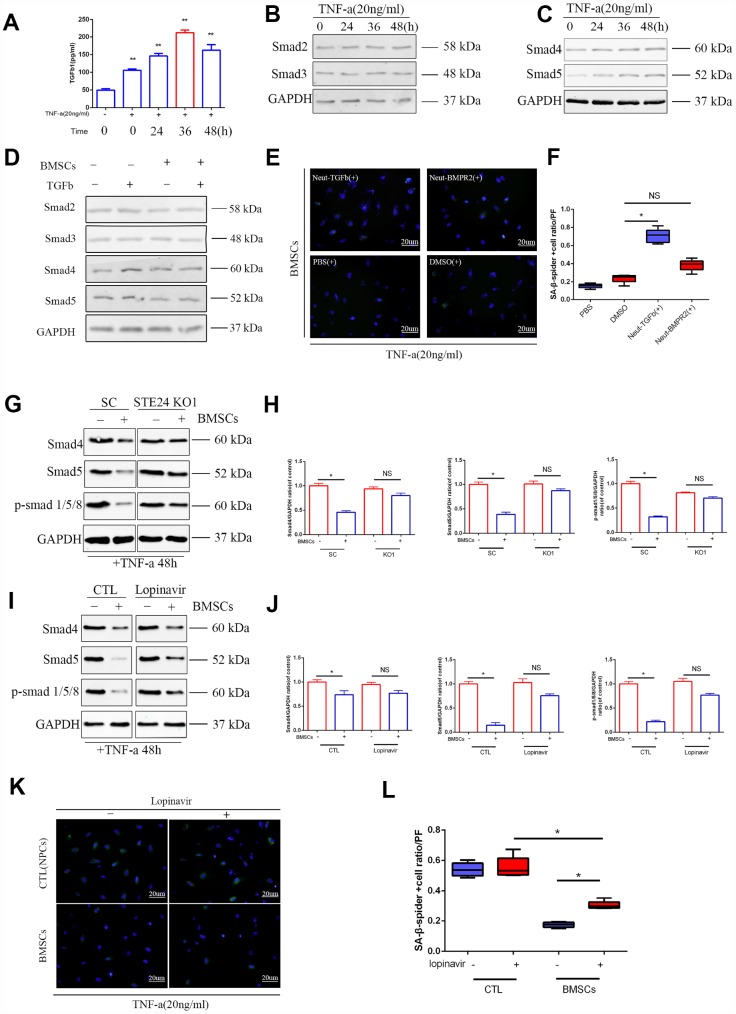
**Effects of ZMPSTE24 on senescence associated TGFβ/Smad signaling pathway.** (**A**) Elisa result of TGFβ1 in senescent NPCs culture supernatant. (**B**–**C**) Protein expressions of Smad2, Smad3, Smad4 and Smad5 under TNF-a inducing were visualized by western blot and quantified by Image J. n=3. (**D**) Protein expression of Smad2, Smad3, Smad4 and Smad5 were visualized by western blot and quantified by Image J. n=3. (**E**–**F**) Spider-β-gal staining of NP cells between different neutralizing groups(Neut-TGFb and Neut-BMPR2). Combined with DAPI staining for nuclei. n=5, Scale bar, 20um. (**G**–**H**) Protein expression of Smad4, Smad5 and p-Smad1/5/8 in NP cells with stable scramble vector (SC) or knockout of ZMPSTE24 were visualized by western blot and quantified by Image J. n=3. (**I**–**J**) Protein expression of Smad4, Smad5 and p-Smad1/5/8 in NP cells with or without BMSCs coculture were visualized by western blot and quantified by Image J. n=3. (**K**–**L**) Spider-β-gal staining of NP cells between different coculture groups(NPCs or BMSCs) with or without lopinavir. Combined with DAPI staining for nuclei. n=5, Scale bar, 20um. Values represent means±S.D. Significant difference between different groups is indicated as *P < 0.05. PF: per field.

The TGFβ pathway plays a critical role in cellular senescence, cell cycle arrest and cell survival by transcriptional induction of p21 and activation of the PI3K-AKT pathway [31]. In previous studies, co-activation of TGFβ and NF-κB signaling promoted cell aging during nucleation of cells [32]. It was also shown that co-culture with MSCs reduced the expression of inflammatory markers in senescent NPCs [33]. We observed that TGFβ1 levels increased in culture supernatants from senescent NPCs up to 36 h after exposure to TNF-α (20 ng/ml) (Figure 5A). In parallel, a gradual increase in Smad2/3/4/5 expression was noted over 48 h upon stimulation with TNF-α (Figure 5B and 5C and Supplementary Figure 1H). These data suggest that the TGFβ pathway is activated by inflammatory stimulation in NPCs. In line with the findings reported above, decreased overall Smad protein levels were measured in NPCs co-cultured with BMSCs (Figure 5D and Supplementary Figure 1I). On the other hand, the rate of SA-β-Gal-positive NPCs was reduced after antibody-mediated neutralization of BMPR2, which binds BMPs and other members of the TGFβ superfamily of cytokines, whereas a TGFβ neutralizing antibody was less effective (Figure 5E and 5F). This suggests that a reduction of BMP signaling is involved in the anti-senescent effects of BMSCs on NPCs.

The involvement of TGFβ signaling on inflammation-induced NPC senescence was further evaluated after ZMPSTE24 downregulation in NPCs, either by CRISPR/Cas9 editing or treatment with lopinavir. Western blot results showed that Smad4/5 and p-Smad1/5/8 expression in NPCs was attenuated upon BMSC co-culturing, and these changes were counteracted by silencing or inhibition of ZMPSTE24 (Figure 5G–5J). Accordingly, SA-β-Gal-staining increased significantly in NPCs from Sene + BMSCs co-cultures after inhibition of ZMPSTE24 with lopinavir (Figure 5K and 5L). These data suggest that co-culture with MSCs represses TGFβ pathway activation and attenuates NPC senescence through upregulation of ZMPSTE24.

### BMSCs delay NPC degeneration in ex-vivo NP explants

**Figure 6:**
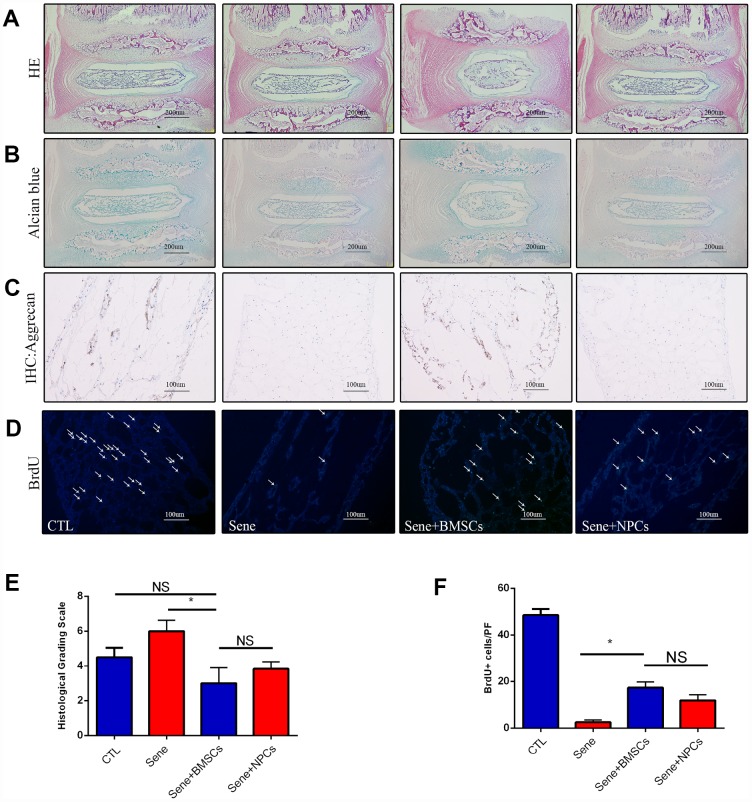
**Staining with HE, Alcian blue, immunohistochemistry of aggrecan and BrdU between CTL, Sene, Sene+BMSCs and Sene+NPCs groups.** n=5, Scale bar, as indicated. Values represent means±S.D. Significant difference between different groups is indicated as *P < 0.05.PF: per field.

To evaluate the anti-senescent effects of MSCs on NPCs under conditions that more closely mimic the in vivo environment, intervertebral disc tissues from SD rats were isolated to generate ex-vivo NP explant cultures. NP tissue structure was assessed through HE and Alcian blue staining in healthy control cultures (CTL), following senescence induction (Sene), in BMSCs co-cultures (Sene + BMSCs), and in co-cultures with normal NPCs (Sene + NPCs). Compared with the other culture conditions, NP explants in Sene + BMSCs co-cultures partially preserved tissue structure and showed also some degree of extracellular matrix condensation. According to Masuda’s histological scoring system [34], average grading in Sene + BMSCs (2.6 ± 0.5) was significantly lower than in Sene (6.2±0.5) and Sene + NPCs (4.5 ± 0.4) (Figure 6A–6E). Histological examination of NP explants in the Sene + BMSCs system revealed that intervertebral discs were relatively intact, and preserved NP structure with obvious cartilage-like staining (Figure 6B). Accordingly, significantly higher aggrecan immunoreactivity was detected in NP explants in Sene + BMSCs co-cultures compared with those in the Sene and Sene + NPCs groups (Figure 6C). These data suggest that MSCs promote the secretion of proteoglycans, stabilizing NP tissue structure in ex-vivo organ cultures. Further evidence that MSCs attenuate inflammatory degeneration of NP was provided by BrdU incorporation experiments, which showed that the rate of BrdU-positive NPCs was significantly higher in Sene + BMSCs than in Sene co-cultures (Figure 6D). These data demonstrate that MSCs can promote normal function and stimulate proliferative activity in NPCs in ex-vivo organ cultures.

**Figure 7:**
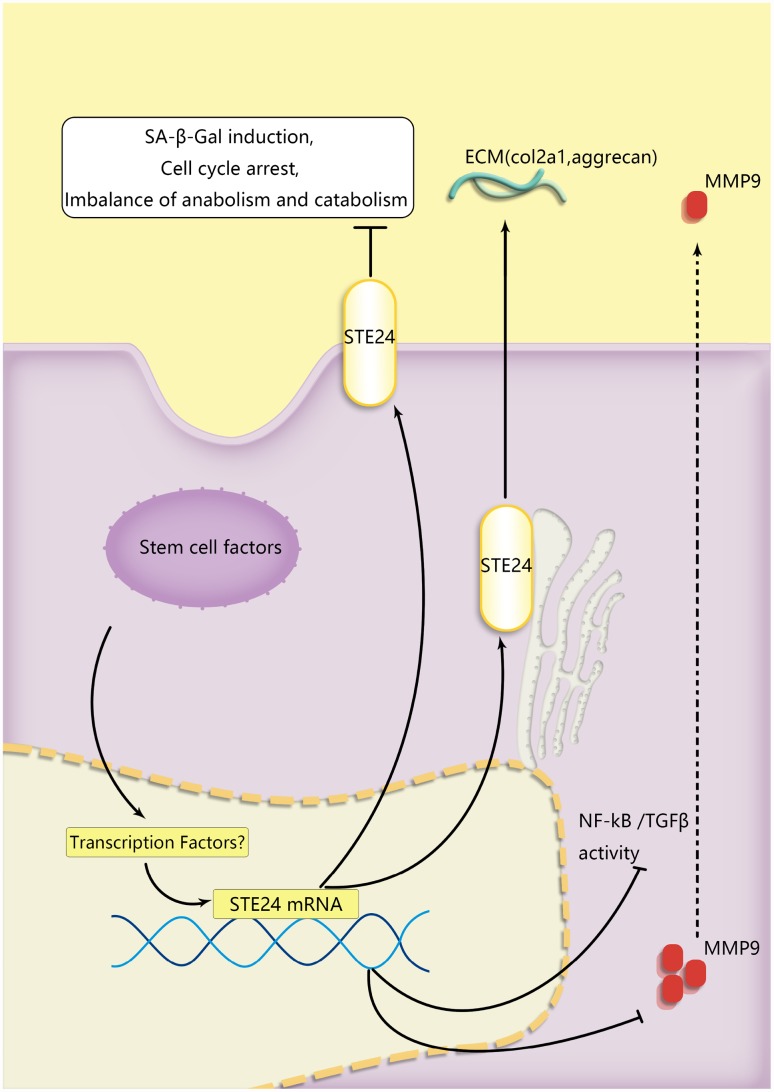
**A schematic diagram of the proposed mechanisms of BMSCs’ protection of senescent NP cells during coculture.** ZMPSTE24 was upregulated to reduce MMP9 to restore NP cells’ functional ECM balance. TGFβ and RelA which were involved in senescent pathway were alleviated during NP cells degeneration.

## DISCUSSION

Despite intense basic research efforts paralleled by the development of various therapeutic strategies, the pathogenesis of IDD has not been completely elucidated. The use of MSCs to treat NP degeneration holds great therapeutic potential as it entails low teratogenicity [35], provides cell growth- and cell survival-promoting factors [36], and has sustained effects [37]. The literature describes examples of feedback regulation between stem cell-like progenitor cells and mature NPCs [38]. For example, Sox9-transfected MSCs were used to treat NP degeneration in rabbits [29], and BMSC-derived exosomes were shown to attenuate IDD in a caudal disc degeneration model in rats [17]. The present work indicated the presence of activated MSCs in the NP of degenerating (Pfirrmann grade III-IV) human intervertebral discs, and showed that inflammation-induced NPC senescence is attenuated, and NPC viability and function partially restored, by MSCs co-culturing. We identified several mechanisms underlying these effects, including an anabolic switch indicated by increased Coll-IIa deposition and decreased MMP9 expression, was well as mitogenic stimulation reflected by increased S phase frequency and decreased expression of the cell cycle inhibitory proteins p16^Ink4a^ and p21. In ex-vivo NP organ cultures, MSCs helped preserve NP tissue integrity. Consistent with other results, no obvious changes were seen in cartilage abundance after Alcian blue staining; however, the expression of proteoglycans (aggrecan) did increase significantly. Importantly, co-culture with MSCs also favored NPC proliferation in NP explants, as shown by BrdU incorporation experiments.

We also explored the molecular basis of NPC senescence and the protective effects of MSC co-culture. The TGFβ pathway is associated with fibrosis, often in association with inflammatory conditions, and previous studies also reported its upregulation during cell senescence [39]. On the other hand, the release of downstream inflammatory factors upon activation of the NF-κB pathway crucially contributes to cartilage degenerative disease [40]. Accordingly, our RNA-seq analysis showed higher overall downregulation of the SASP phenotype, which promotes NP senescence and cartilage degradation, in NPCs co-cultured with BMSCs rather than with normal NPCs. Meanwhile, co-culture with BMSCs promoted the expression of the premature aging-related gene ZMPSTE24. In this regard, the differential expression of the transcription factor genes sox5 and ccl27(Figure 3H) and the involvement of the NF-kB pathway suggest that BMSCs may play a transcriptional protective effect [41, 42] on senescent NPCs by upregulating ZMPSTE24. To verify the above results, ZMPSTE24 was alternatively knocked down and overexpressed in NPCs. ZMPSTE24 overexpression decreased the expression and nuclear translocation of RelA, indicating reduced NF-κB pathway activation in TNF-α-stimulated NPCs. Meanwhile, downregulation of Smad4/5 and pSmad1/5/8 indicated decreased TGFβ signaling activation in senescent NPCs, both after BMSC co-culturing and following ZMPSTE24 overexpression, while ZMPSTE24 silencing or inhibition abrogated these effects. Therefore, our data suggests that MSC-induced upregulation of ZMPSTE24 contributes to inhibition of inflammatory and degenerative processes in NPCs.

Cartilage degenerative diseases have an inflammatory component involving increased expression of pro-inflammatory factors such as TNF-α, IL-1β, and IL-6. Previous research demonstrated that stem cells can mitigate cell dysfunction and premature senescence through modulation of major pro- and anti-inflammatory pathways [43]. This study focused on NPCs but did not specifically addressed gene expression and functional changes in MSCs. Therefore, future studies are needed to assess the molecular pathway(s) involved in the MSC-induced upregulation of ZMPSTE24 in NPCs, and on BMSCs’ paracrine regulation of senescence-related pathways in degenerative disc disease.

Past research provided evidence for partial restoration of NPCs’ normal phenotype by conventional two-dimensional co-culture with MSCs [16]. Our MSC 3D co-culture system proved to be superior, as it extended previous findings and unmasked additional effects (Supplementary Figure 2E and 2F). Unlike previous research using 2D co-cultures, MSCs in 3D scaffolding material (sodium alginate) can not only actively maintain a microenvironment that propitiates cell growth, but can also contain more viable cells (by about an order of magnitude) due to the expansion of the supporting surface area. In addition, the 3D scaffold architecture used here ensures cellular viability and does not overtly interfere with normal BMSC properties. Compared with 2D co-culture conditions, BMSCs in 3D cultures retain the advantages of conventional co-culture but avoids direct contact between stem cells and target cells [44].

Several limitations exist in current invasive treatments for IDD, such as the host’s inability to support the viability of numerous cell transplants, and complications such as infection and discitis. Thus, new hopes for IDD and age-related diseases are being placed on research developments centered on small molecule-based therapies and MSC-based regenerative medicine. The identification of ZMPSTE24 as an important MSC-induced modulator of NPC survival and function guarantees future studies to confirm its clinical significance. In addition, optimization of the 3D MSC co-culture system and molecular identification of factors responsible for MSC/NPC cross-talk also require further research.

## MATERIALS AND METHODS

### Degenerative intervertebral disc specimens

Degenerative intervertebral discs were obtained from 17 patients (9 males and 8 females; average age: 56 ± 4 years; Pfirrmann grade range: III-IV; lumbar disc range: L2-S1). NP samples were subjected to immunohistochemistry analysis and primary cell isolation (Table 1). Patients’ samples were obtained after institutional review board approval by the Translational Science Research Review Committee of the Ninth People’s Hospital, Shanghai Jiaotong University School of Medicine.

**Table 1:** Clinical case Pfirrmann Garde information.

**Donor ID**	**Sex**	**Age**	**Site of LDH**	**Pfirrmann Grade**
*1	Male	55	L3/L4	III
*2	Male	56	L3/L4	III
*3	Male	57	L4/L5	III
*4	Male	55	L5/S1	IV
*5	Female	53.5	L3/L4/L5	III
*6	Female	56	L2/L3	IV
*7	Male	57	L5/S1	IV
*8	Female	59	L3/L4	IV
*9	Male	58	L3/L4/L5	IV
*10	Female	58.5	L5/S1	III
*11	Male	51	L4/L5	IV
*12	Male	53	L5/S1	IV
*13	Female	58.5	L4/L5	III
*14	Female	54.5	L5/S1	IV
*15	Female	57	L4/L5	IV
*16	Male	52	L4/L5	IV
*17	Female	55	L4/L5/S1	III

Note. LDH: lumber disc herniation.

### Primary cell culture

### Isolation of primary nucleus pulposus cells (NPCs)

Human NP tissues (from intervertebral discs L2-S1) were isolated under a microscope. Tissue pieces were cut into 0.1 x 0.1 mm^3^ fragments in a sterile Petri dish and blood was rinsed with sterile PBS. NP tissues from male SD rats (4 weeks old) were obtained from caudal discs (C3-C6) after euthanasia with pentobarbital (35 mg/kg), and transferred to 0.25% type II collagenase for overnight digestion in a CO_2_ incubator. After centrifugation, the cells were resuspended and seeded in monolayer culture with DMEM containing 10% fetal bovine serum (FBS, Gibco) and 1% penicillin-streptomycin (Sigma). Cells were incubated at 37°C, 21% O_2_, and 5% CO_2_. All the experimental procedures followed the Guide for the Care and use of Laboratory Animals of the National Institutes of Health and were approved by the Animal Care and Animal Ethics Committee of the Shanghai Jiaotong University School of Medicine.

### Isolation of bone marrow-derived mesenchymal stem cells (BMSCs)

BMSCs were isolated from femurs and tibias of male SD rats (4-5 days old) following euthanasia with pentobarbital (20 mg/kg). Bone marrow cells were flushed out with a syringe (1 ml) into flasks (Corning), and plated in monolayer culture with aMEM containing 10% FBS at 37°C, 21% O_2_, and 5% CO_2_.

### Senescent NPCs and 3D CAGB co-culture model

### NPC senescence model

Inflammation-induced senescence was induced in passage 2 rat NPCs by treatment with 20 ng/ml TNF-α over 48 hs.

### 3D co-cultures

The 3D co-culture system was based on the alginate hydrogel method (Calcium Alginate Gel Balls; CAGB). BMSCs or normal (non-TNF-α-treated) NPCs were thoroughly mixed in a 2% sodium alginate solution at a density of 10^7^ cells/ml, and delivered at a rate of 100 μl per drop onto culture dishes containing a 0.15% CaCl_2_ cross-linking solution to form spherical beads. The hydrogel spheres were next transferred to a 0.2% CaCl_2_ solution for about 5 min to complete the gelation process. After rinsing twice with sterile PBS, alginate hydrogel spheres complexed with BMSCs or normal NPCs were collected for subsequent experiments. Alginate-only (cell-free) CAGB were used as co-culture control.

### Cell viability analysis

The viability of NPCs in different co-culture conditions was evaluated with the CCK-8 assay (Dojindo, Japan). NPCs were seeded in 96 well plates (5,000 cells/well) for 12, 24, or 48 h, and washed with PBS before addition of CKK-8 (10 μl/well) in complete DMEM (100 μl). DMEM without CCK-8 was used as blank. Optical density (OD) at 450 nm was recorded using a microplate reader (BIOTEK, USA). The ODs for each treatment group were subtracted from corresponding ODs from blank samples to exclude background interference.

### Senescence-associated-ß-galactosidase activity assay

For SA-β-Gal staining in fixed cells, NPCs (5×10^5^ cells/well) were seeded in 6-well plates and co-cultured with different CAGB groups for 2 days. At the end of co-culturing, NPCs were washed twice with PBS and fixed with 4% paraformaldehyde for 15 min. Samples were washed with PBS and then stained using a SA-β-Gal staining kit (Sigma). SA-β-Gal-positive cells were counted under light microscopy, and data expressed as the percentage of positive cells using Image J software.

For SA-β-Gal staining in living cells, NPCs (10^5^ cells/well) were grown on microscope cover glasses in 6-well plates, and co-cultured with different CAGB groups with or without the ZMPSTE24 inhibitor lopinavir (20 μM) for 2 days. After three PBS washes, cultured cells were further incubated for 1 h in the presence of 1 ml bafilomycin A1 (to inhibit endogenous β-galactosidase activity). Next, fluorogenic β-galactosidase detection was carried out by adding 1 ml of SPiDER-β-Gal staining solution (Dojindo, Japan) to each well for an additional 30 min. All these steps were carried out on an incubator at 37°C/5% CO_2_. The cells were then washed, and DAPI (Beyotime, China) was used to stain nuclei for 5 min at room temperature. Anti-TGFβ1 and anti-BMPR2 antibodies (10 μg/ml; Abcam) were used for neutralization experiments before SA-β-Gal staining. Fluorescence microscopy (Olympus IX71) was used to record SA-β-Gal-positive cells, and quantification was performed using Image J software.

### Cell cycle analysis

NPCs seeded in 6-well plates (10^5^ cells/well) co-cultured with different CAGB-cell complexes for 24 h. NPCs were then collected, washed with PBS 3 times, centrifuged, and fixed in 70% cold ethanol for 24 h at 4°C. Cells were then stained with propidium iodide (PI; Thermo Fisher Scientific, Waltham, MA, U.S.A) and cell cycle distribution analyzed by flow cytometry. In separate experiments, co-cultured NPCs were synchronized at G1 phase by incubation with lovastatin for 34 h and then cultured in medium containing 2 mM mevalonic acid [45]. At indicated periods thereafter, cells were harvested and stained with PI for cell cycle analysis. Data visualization and analysis were performed with FlowJo software (Version 10, FlowJo, LLC).

### Colony formation assay

Colony formation assays were performed using double-layer soft agar in 12-well plates (top layer 0.35% agar; bottom layer 0.7% agar). Cells were seeded into 12-well plates in appropriate media and cultured at 37°C for 15–20 d, and resulting cell colonies were stained and counted.

### Quantitative real-time PCR

Total RNA was isolated from NPCs using a total RNA preparation kit (Axygen, NY, U.S.A) according to the manufacturer’s protocol. A cDNA synthesis kit (TAKARA, Dalian, China) was used for reverse transcription to prepare first-strand cDNA, and gene expression was measured by quantitative PCR (Thermo Fisher QuantStudio 6 Flex system, USA) using TaqMan and SYBR green protocols. Relative changes in gene expression were assessed with the 2^(–ΔΔCt) method. GAPDH was used as the endogenous control.

Primers for p16^Ink4a^, p21, p53, and GAPDH were designed using Primer3web version 4.1.0 (http://primer3.ut.ee/): p16, (F) 5′- TGGACTTGGAGGA GAGAACC -3′ (R) 5′- CATTGACAGACGACGATG ATG -3′; p21, (F) 5′- ACTTCGGCATCAGTGGACA -3′, (R) 5′- AGACATCAGAGCGGACATCA -3′; p53, (F) 5′- CTCCTCTCCCCAGCAAAAGA -3′, (R) 5′- GTAGACT GGCCCTTCTTGGT -3′; GAPDH, (F) 5′- AACGACCC CTTCATTGACCT -3′, (R) 5′- ATGTTAGTGGGGTCT CGCTC -3′.

### Immunohistochemistry and ex-vivo proliferation analysis

After surgery, disc tissues from patients or rats were fixed in 4% paraformaldehyde for 48 h, sectioned, and stained with Alcian blue or hematoxylin and eosin. For immunohistochemistry, tissue slides were incubated in blocking buffer at room temperature for 30 min, and a primary antibody against anti-aggrecan (Cell Signaling Technology, 1:100) was applied at 4°C overnight. Images were analyzed with Image J software.

Ex-vivo cell proliferation was assessed on intervertebral discs by the BrdU method according to manufacturer’s instructions (Biovision). Briefly, BrdU (0.03 μg/ml) was added into the discs’ media on the 6st day post-excision, washed out with PBS after 8 h, and 4% paraformaldehyde was added for 48 h. Subsequently, tissue sections were obtained after decalcification and paraffin embedding. BrdU incorporation was detected by fluorescence imaging after DAPI staining and analyzed with Image J software.

### Immunofluorescence

NPCs (5×10^5^ cells/well) were seeded in 6-well plates, co-cultured with different CAGB-cell complexes for 2 days, washed twice with PBS and fixed with 4% paraformaldehyde for 15 min. Following permeabilization with 0.1% TritonX-100 in PBS for 10 min, cells were blocked with 5% bovine serum albumin (BSA) for 1 h at 37 °C, rinsed with PBS, and incubated with PBS-diluted primary antibodies: collagen IIa (1:200), MMP9 (1:200), ZMPSTE24 (1:300), or RelA (1:200) in a humid chamber overnight at 4 °C. On the next day the samples were washed and incubated with Alexa Fluor®488- or Alexa Fluor®594-conjugated secondary antibodies (1:500) for 1 h at room temperature, and labeled with DAPI for 5 min. Images were recorded using a fluorescence microscope and analyzed with Image J software.

### Ex-vivo disc organ co-culture model

Five male SD rats (4 weeks old) were anesthetized with pentobarbital (35 mg/kg) for isolation of intervertebral discs from caudal C3-C6 vertebrae. Discs parts were divided into 4 groups and placed in 6-well plates for co-culture with three different CAGB-cell systems (i.e BMSC, normal NPCs or cell free). Twenty CAGBs were used in each co-culture. In brief, tissue explants were slowly covered with ~3.5 ml of DMEM, and then incubated at 37°C with 5% CO_2_. 24h later, tissues were incubated for about 2 days with TNF-α (200 ng/ml) [46], and BMSC-CAGB or NPC-CAGB complexes were added to generate co-cultures. For NPC proliferation assessment, TNF-α (200 ng/ml) was administered as above and co-cultures maintained for an additional 7 days. BrdU (0.03 μg/ml) was added on day 6 and its incorporation assessed on day 7.

### Lentiviral transduction

NPCs were transduced with lentiviral vectors (pMD2.G and psPAX2) containing CRISPR/Cas9 constructs to knockdown (KO) ZMPSTE24 (KO1: ACCAGAGTTA GAACAGATCATGG, KO2: CTGGTCAGGACTCTA TTCAGAGG, KO3: GGAGAAGCGAATCTTCGGGG CGG) or a scrambled control. For ZMPSTE24 knockdown, cells were selected in 5 μg/mL blasticidin S (Yeasen, 60218ES10, China) for 48 h and maintained thereafter in 2.5 μg/mL blasticidin S. For ZMPSTE24 overexpression (OE), the cells were selected in 3 μg/mL puromycin (Sigma-Aldrich) for 48 h and maintained thereafter in 1 μg/mL puromycin. In both cases, selection antibiotics were first applied 72 h after transduction. Western blotting was used to confirm KO and OE efficiency.

### Protein extraction

NPC proteins were extracted by RIPA lysis buffer with a protease inhibitor and EDTA (Roche, Grenzach, Germany). Nuclear and cytoplasmic proteins were extracted with a Nuclear and Cytoplasmic Protein Extraction Kit (Sangon, Shanghai, China), and quantified using a BCA Protein Assay Kit (Sangon, Shanghai, China).

### Western blotting

Equal amounts of protein (40 ng) were separated by 12.5% or 10% SDS-PAGE and transferred to 0.22 μm polyvinylidene fluoride (PVDF) membranes (Merck Millipore, CA, U.S.A). After blocking with 5% fat-free milk for 2 h, the membranes were incubated overnight at 4 °C with primary antibodies (from Cell Signaling Technologies) against P16^Ink4a^ (1:500), p21 (1:500), RelA (1:500), ZMPSTE24 (1:500), Smad2 (1:1000), Smad3 (1:1000), Smad4 (1:1000), Smad5 (1:1000), p-Smad1/5/8 (1:1000), β-actin (1:5000), lamin-B (1:5000), and GADPH (1:5000). Next, an anti-rabbit IgG antibody conjugated to IRDye 800CW (Cell Signaling Technologies) was applied for 1 h at RT, and immunoreactive bands visualized using an Odyssey infrared imaging system (LI-COR). Signal quantification by normalization to endogenous control was done with Image J software.

### RNA sequencing

Total RNA from 2-day-old NPCs harvested after different co-culture conditions was extracted using the RNeasy Mini kit (Qiagen, Valencia, CA, USA) following the manufacturer’s protocol. RNA integrity was evaluated using an Agilent 2100 Bioanalyzer (Agilent Technologies, Santa Clara, CA, USA). RNA sequencing was carried out by Solexa high-throughput sequencing service (Oebiotech, Shanghai, China).

### Statistical analysis

The experiments were performed at least 3 times. Data from all experiments are presented as mean ± S.D. Statistical analyses were performed using SPSS statistical software program 20.0 and figures were prepared using GraphPad Prism 6.0 software. Data were analyzed by one-way analysis of variance (ANOVA) followed by Tukey’s test for comparison between control and treatment groups. *P* < 0.05 was considered significant.

### Accession number

The sequencing data have been deposited in the NCBI Sequence Read Archive (SRA) database under the accession code SRR10251586.

## Supplementary Material

Supplementary Figures
