# Supplementary material for: Bone marrow-derived mesenchymal stem cells in three-dimensional co-culture attenuate degeneration of nucleus pulposus cells
Source: Aging (Albany NY). 2019 Oct 30;11(20):9167–87. doi: 10.18632/aging.102390 (PMC6834418; doi:10.18632/aging.102390)
Supplement: Supplementary Figures [file aging-11-102390-s001.pdf]

SUPPLEMENTARY FIGURES

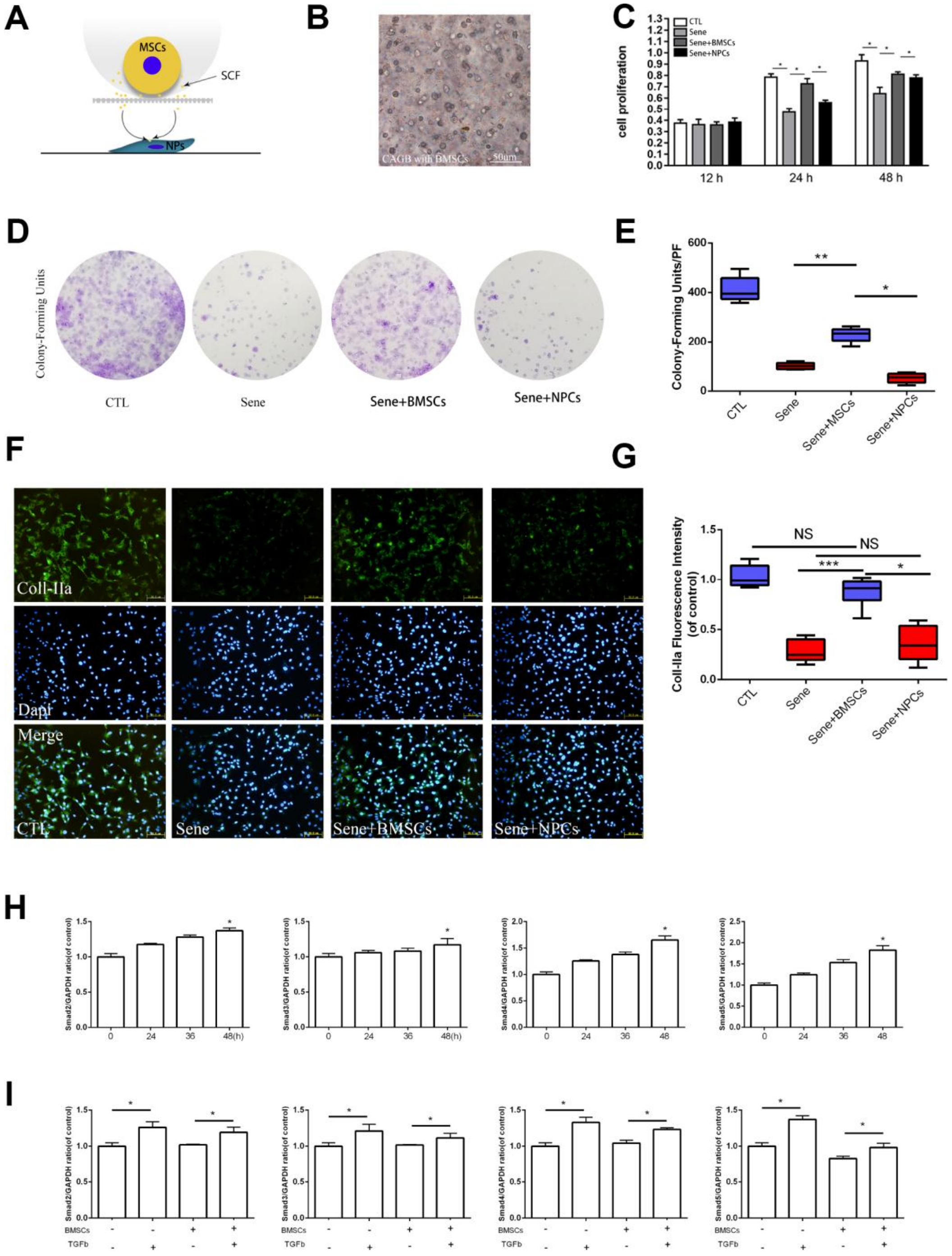

Supplementary Figure 1. (**A**) Co-culture schematic diagram, the possible effect of stem cell paracrine on nucleus pulposus cells (**B**) Bone marrow mesenchymal stem cells encapsulated in sodium alginate pellets under light microscope, Scale bar, 50um (**C**) Proliferative rate of nucleus pulposus cells in different groups. (**D**–**E**) Nucleus pulposus cells cloning ability varies in different groups. (**F**–**G**) Immunofluorescence staining of Col-IIa combined with DAPI staining for nuclei. n=5, Scale bar, 50um. Col-IIa fluorescence intensities were determined by using Image J software. (**H**) Protein expressions of Smad2, Smad3, Smad4 and Smad5 under TNF-a inducing were quantified by Image J. n=3. (**I**) Protein expression of Smad2, Smad3, Smad4 and Smad5 were quantified by Image J. n=3. Values represent means±S.D. Significant differences between different groups are indicated as *P < 0.05, **P < 0.01, ***P < 0.001. PF: per field.

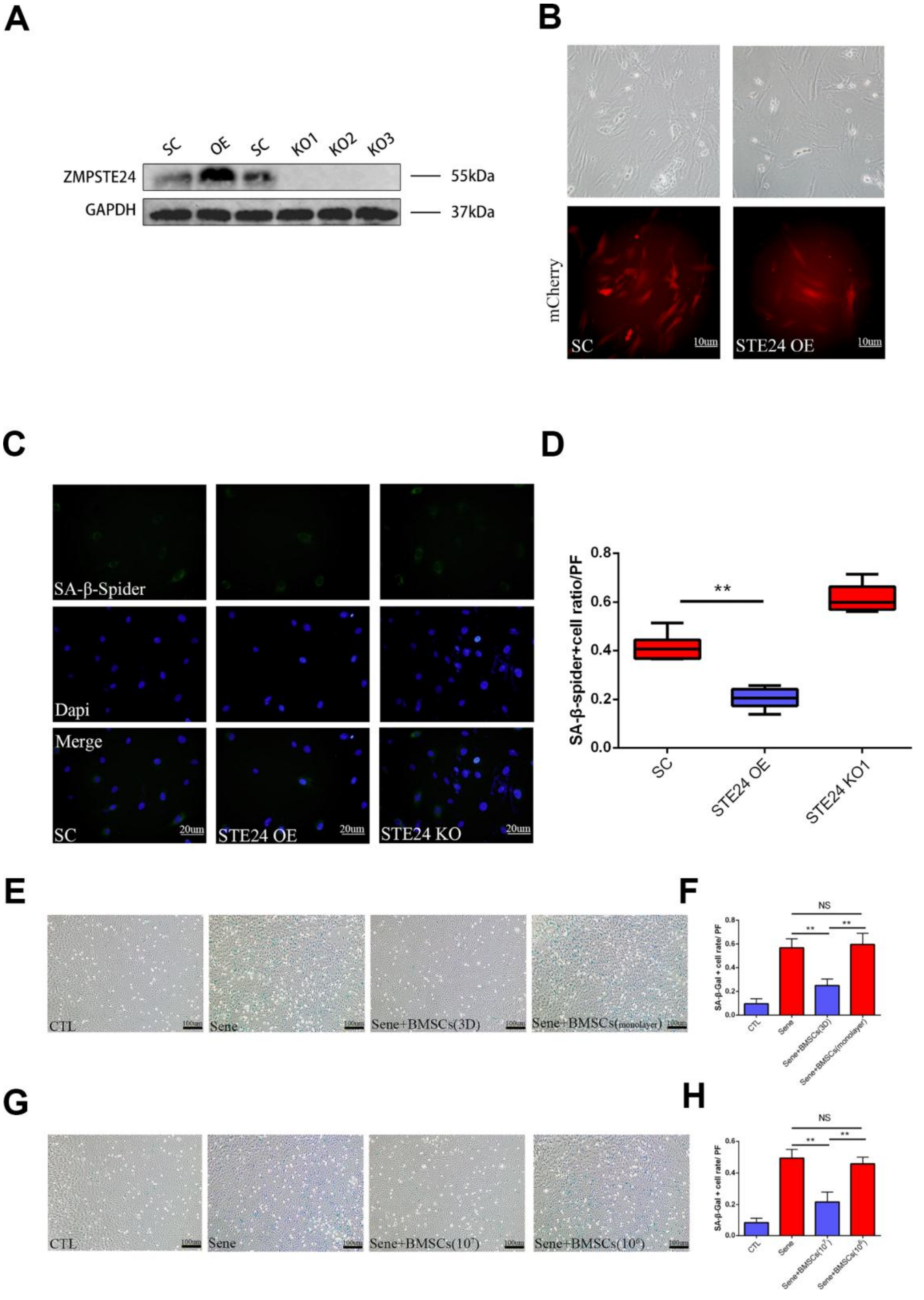

Supplementary Figure 2. (**A**) The ZMPSTE24 overexpressed and knocked out in nucleus pulposus cells were verified by western blots. (**B**) Red fluorescence(mCherry) excited by fluorescence microscopy of nucleus pulposus cells overexpressing ZMPSTE24 (**C**–**D**) Different positive rates of senescence staining (Spiderβgal) stimulated by TNF-a between overexpression and knockout of ZMPSTE24. Combined with DAPI staining for nuclei. n=5, Scale bar, 20um. (**E**–**F**) SA-β-Gal staining of senescent NP cells after 2 days coculture of normal NP cells +blank calcium alginate gel balls(CTL), senescent NP cells+ blank calcium alginate gel balls(Sene), senescent NP cells +calcium alginate gel balls with BMSCs(Sene +BMSCs,3D), senescent NP cells +BMSCs in 2D coculture model(Sene +BMSCs,2D), n=3, Scale bar, 50um. SA-β-Gal positive cell rate between CTL, Sene, Sene +BMSCs(3D) and Sene + BMSCs(2D) groups were determined by using Image J software. (**G**–**H**) SA-β-Gal staining of senescent NP cells after 2 days coculture of normal NP cells +blank calcium alginate gel balls(CTL), senescent NP cells+ blank calcium alginate gel balls(Sene), senescent NP cells +calcium alginate gel balls with BMSCs(107/ml)(Sene +BMSCs,107), senescent NP cells + calcium alginate gel balls with BMSCs(106/ml)(Sene +BMSCs, 106), n=3, Scale bar, 50um. SA-β-Gal positive cell rate between CTL, Sene, Sene +BMSCs(107) and Sene + BMSCs(106) groups were determined by using Image J software. Values represent means±S.D. Significant differences between different groups are indicated as **P < 0.01. PF: per field.

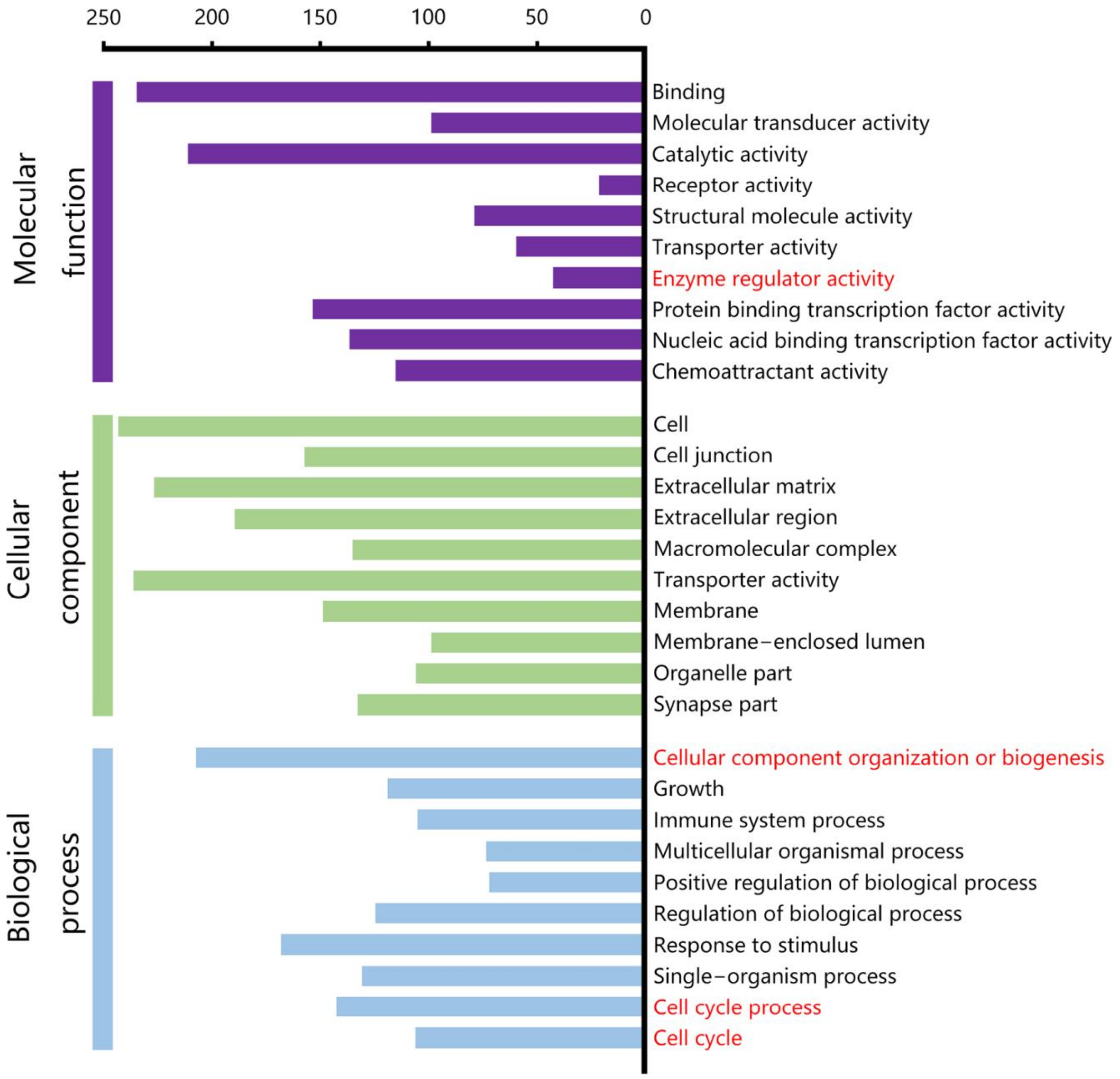

Supplementary Figure 3. Functional characterization of novel candidate identified by differential gene log2FoldChange > 2 between BMSCs and NPCs coculture groups. The top ten GO terms with the smallest P value in the indicated categories are shown (Fisher's exact test, one tailed). The number of proteins for each individual term is also shown. Related to mitosis are marked in red.
